# Supplementary material for: Supervised Myoelectrical Hand Gesture Recognition in Post-Acute Stroke Patients with Upper Limb Paresis on Affected and Non-Affected Sides
Source: Sensors (Basel). 2022 Nov 11;22(22):8733. doi: 10.3390/s22228733 (PMC9692557; doi:10.3390/s22228733)
Supplement: Supplementary file 1 [file sensors-22-08733-s001.zip › sensors-1947155-supplementary.pdf]

sensors

MDPI

Article

Supervised Myoelectrical Hand Gesture Recognition in Post-Acute Stroke Patients with Upper Limb Paresis on Affected and Non-Affected Sides

Alexey Anastasiev [1], Hideki Kadone [2,*], Aiki Marushima [3], Hiroki Watanabe [3], Alexander Zaboronok [3], Shinya Watanabe [3], Akira Matsumura [4], Kenji Suzuki [5], Yuji Matsumaru [3] and Eiichi Ishikawa [3]

[1] Department of Neurosurgery, Graduate School of Comprehensive Human Sciences, University of Tsukuba, 1-1-1 Tennodai, Tsukuba 305-8575, Ibaraki, Japan
[2] Center for Cybernics Research, Faculty of Medicine, University of Tsukuba, 1-1-1 Tennodai, Tsukuba 305-8573, Ibaraki, Japan
[3] Department of Neurosurgery, Faculty of Medicine, University of Tsukuba, 1-1-1 Tennodai, Tsukuba 305-8575, Ibaraki, Japan
[4] Ibaraki Prefectural University of Health Sciences, 4669-2 Amicho, Inashiki 300-0394, Ibaraki, Japan
[5] Center for Cybernics Research, Artificial Intelligence Laboratory, Faculty of Engineering Information and Systems, University of Tsukuba, 1-1-1 Tennodai, Tsukuba 305-8573, Ibaraki, Japan
* Correspondence: kadone@ccr.tsukuba.ac.jp

Supplementary Materials:

Affected and non-affected side feature vector contribution for NP19 and P19 datasets.

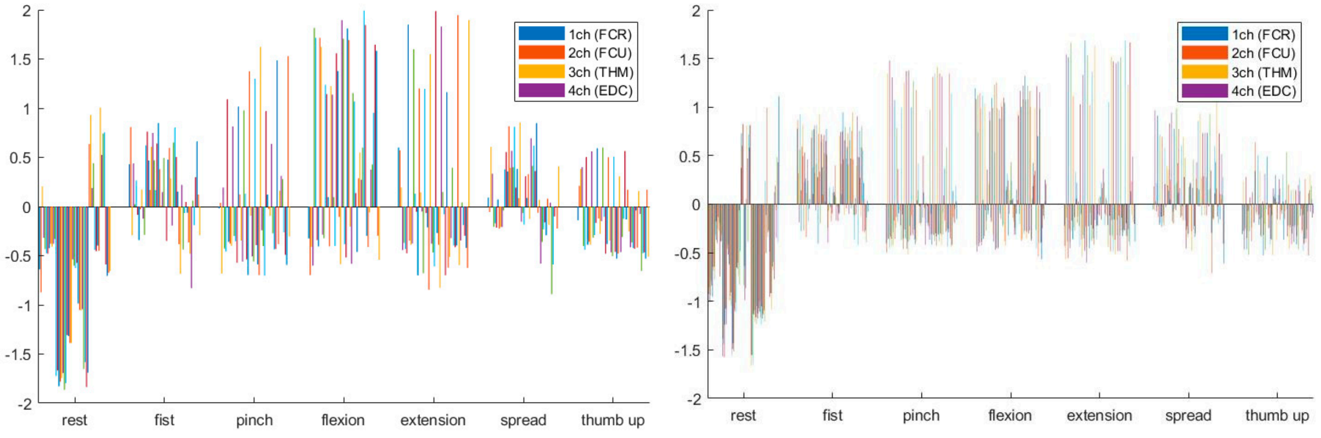

(**a**) (**b**)

Figure S1. This is the vector feature-per-channel contribution for NP19 **(a)** and P19 **(b)** acute stroke data using ASF-14NP feature set for non-affected side, and ASF-24P for the affected side.

Confusion matrix charts of possible random gesture combinations in NP19 using SVM.

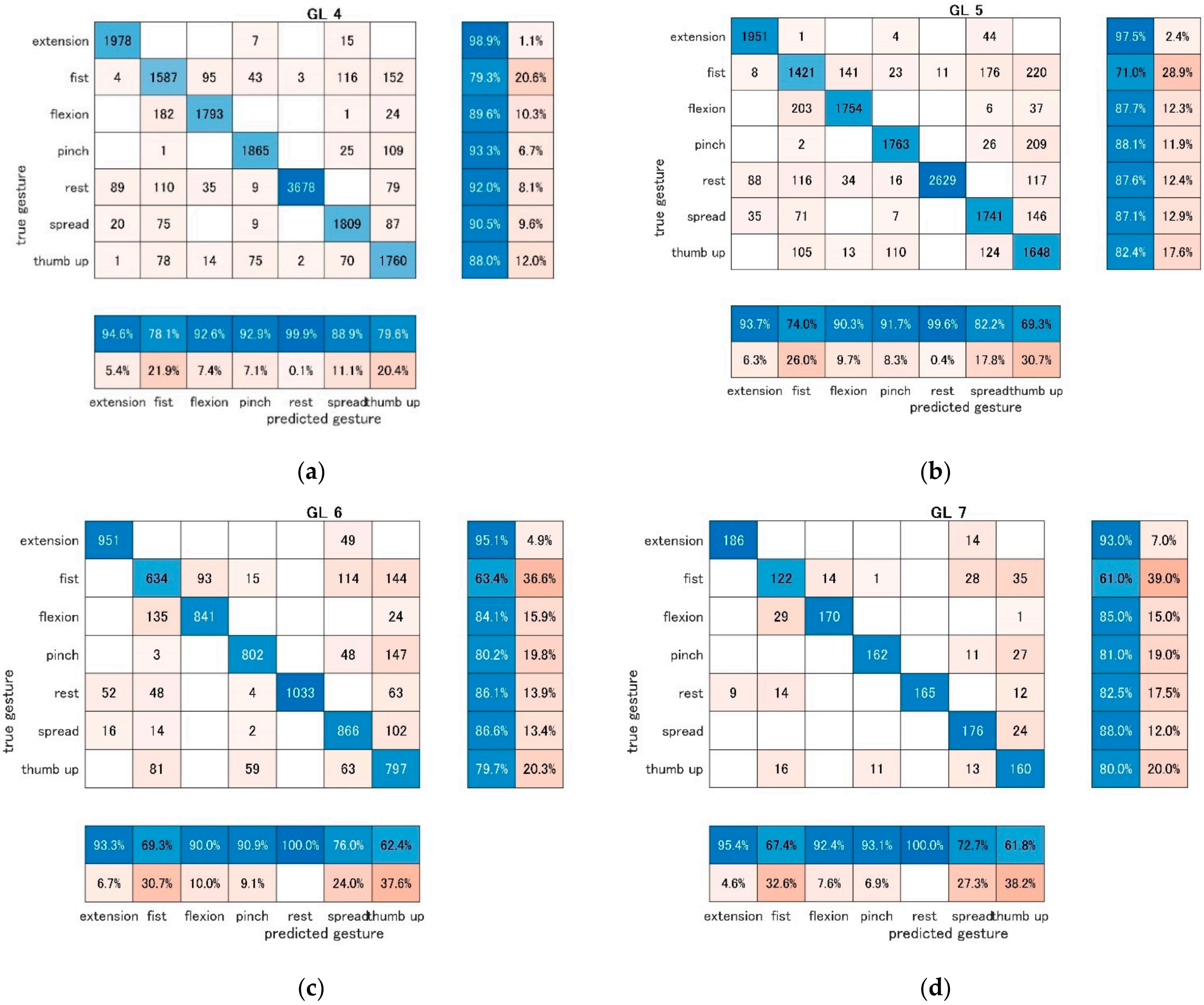

Figure S2. This is non-paretic cross-validation confusion matrix charts with predicted on x-axis and true gesture labels on y-axis which return accuracy rate value using SVM on the non-affected side: (**a**) all possible combinations (20) withing 4 gesture labels (GL4) with the total mean accuracy rate of 95.18%; (**b**) all possible combinations (15) withing 5 gesture labels (GL5) with the total mean accuracy rate of 94.41%; (**c**) all possible combinations (6) withing 6 gesture labels (GL6) with the total mean accuracy rate of 94.28%; (**d**) one possible combination withing 7 gesture labels (GL7) with the total mean accuracy rate of 94.73%. PCA has not been applied for ASF-14NP feature set.

Confusion matrix charts of possible random gesture combinations in NP19 using LDA.

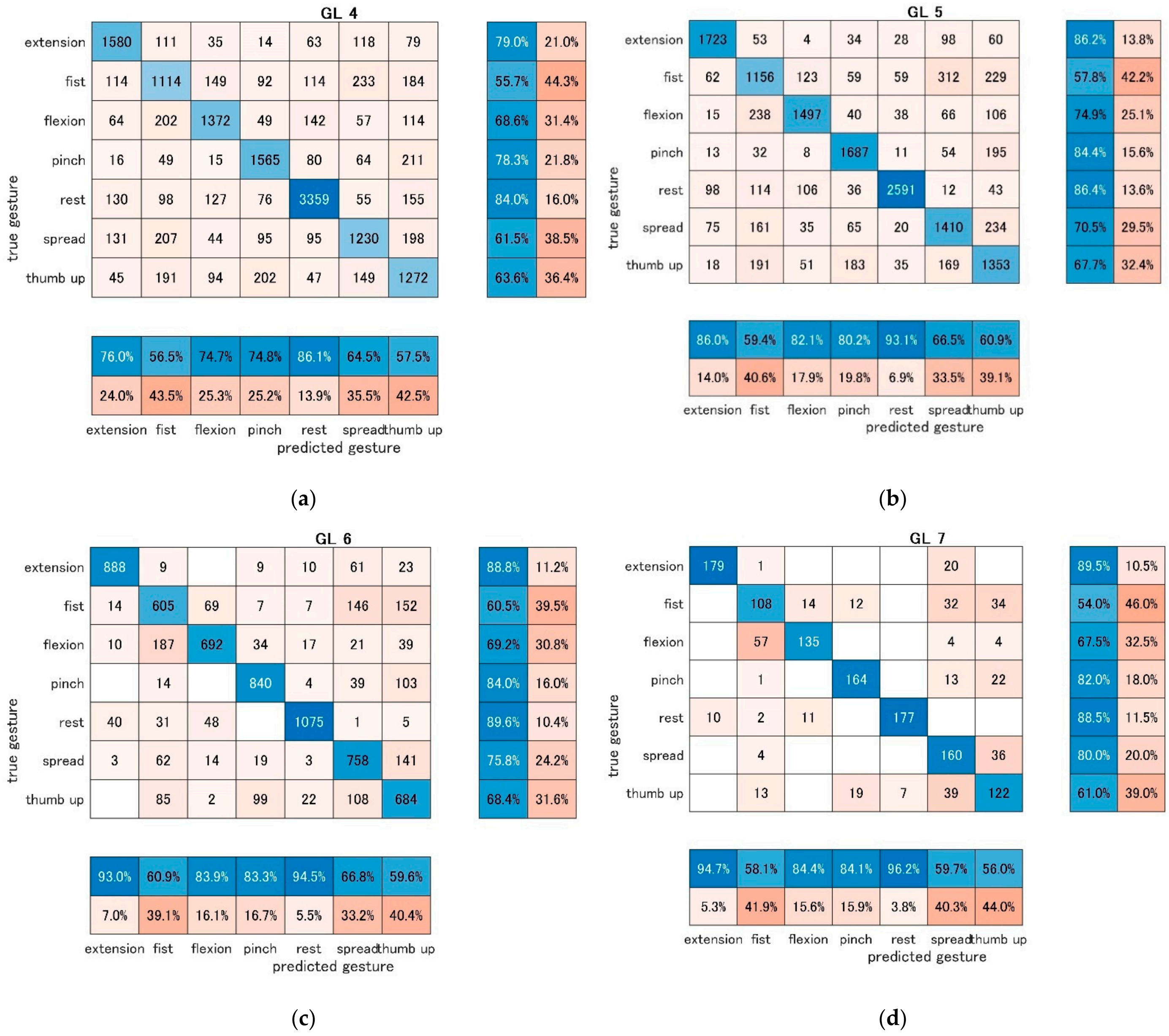

Figure S3. This is non-paretic cross-validation confusion matrix charts with predicted on x-axis and true gesture labels on y-axis which return accuracy rate value using LDA on the non-affected side: (**a**) all possible combinations (20) withing 4 gesture labels (GL4) with the total mean accuracy rate of 88.40%; (**b**) all possible combinations (15) withing 5 gesture labels (GL5) with the total mean accuracy rate of 91.30%; (**c**) all possible combinations (6) withing 6 gesture labels (GL6) with the total mean accuracy rate of 92.49%; (**d**) one possible combination withing 7 gesture labels (GL7) with the total mean accuracy rate of 93.27%. PCA has not been applied for ASF-14NP feature set.

Confusion matrix charts of possible random gesture combinations in NP19 using k-NN.

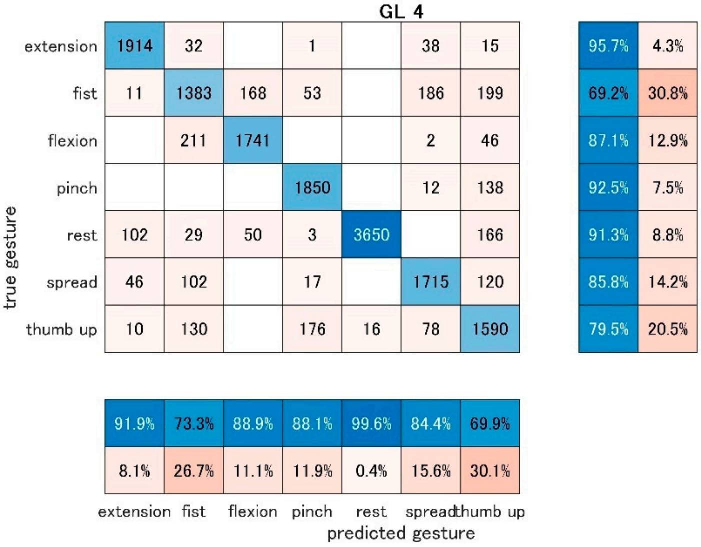

(a)

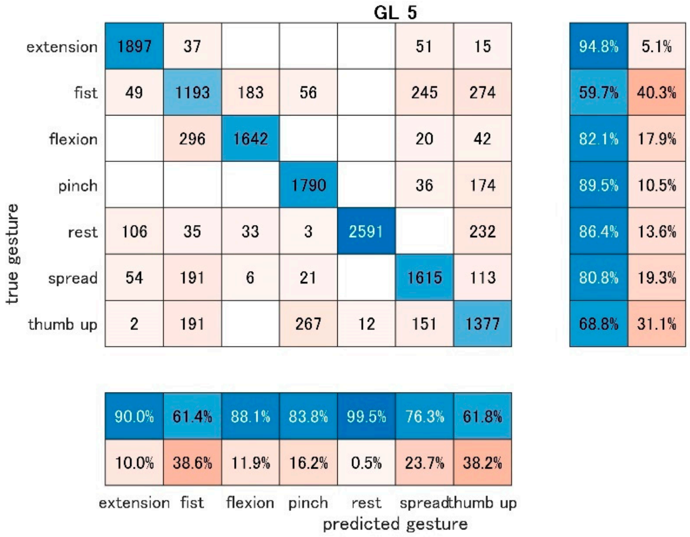

(b)

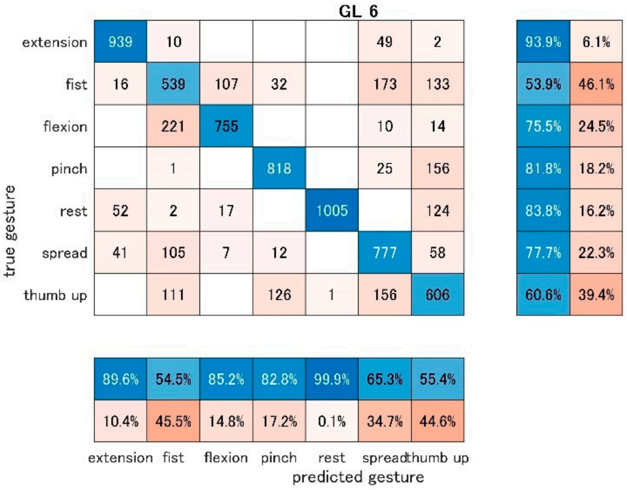

(c)

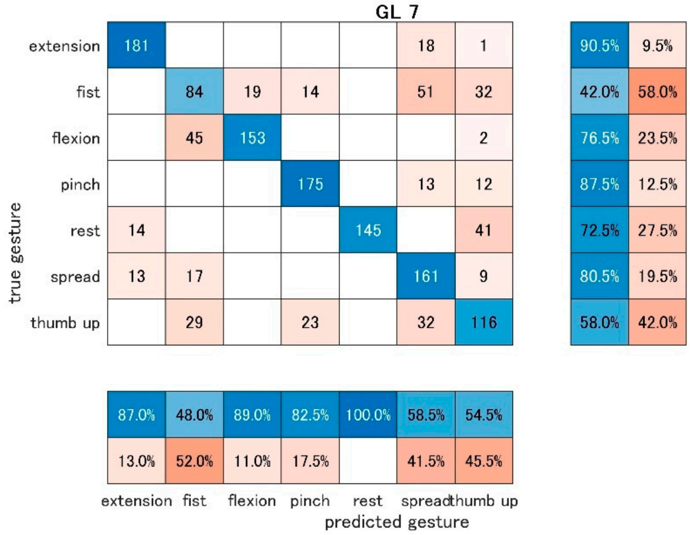

(d)

Figure S4. This is non-paretic cross-validation confusion matrix charts with predicted on x-axis and true gesture labels on y-axis which return accuracy rate value using k-NN on the non-affected side: (**a**) all possible combinations (20) withing 4 gesture labels (GL4) with the total mean accuracy rate of 93.42%; (**b**) all possible combinations (15) withing 5 gesture labels (GL5) with the total mean accuracy rate of 92.34%; (**c**) all possible combinations (6) withing 6 gesture labels (GL6) with the total mean accuracy rate of 91.85%; (**d**) one possible combination withing 7 gesture labels (GL7) with the total mean accuracy rate of 91.71%. PCA has not been applied for ASF-14NP feature set.

Confusion matrix charts of possible random gesture combinations in P19 using SVM.

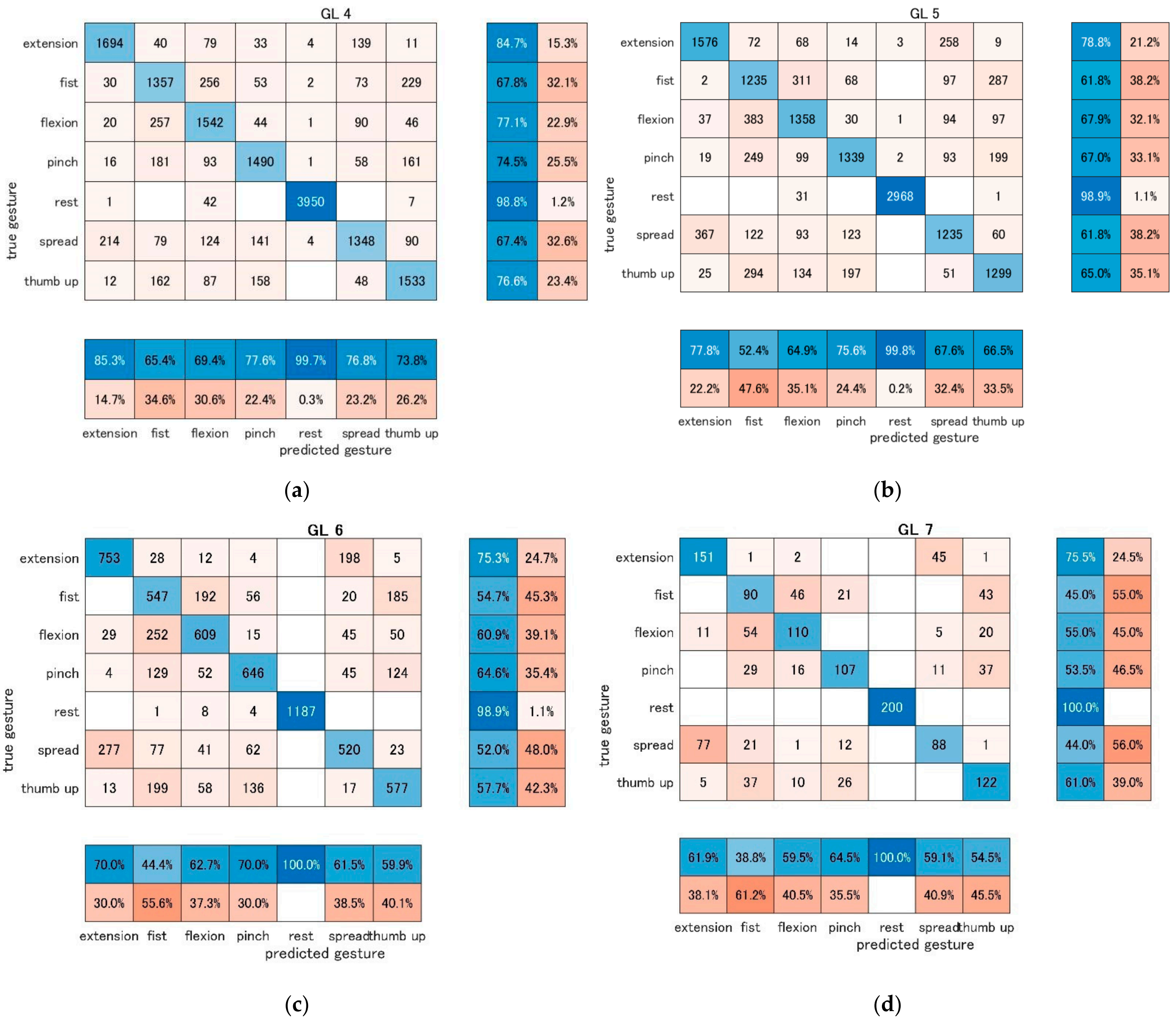

Figure S5. This is paretic cross-validation confusion matrix charts with predicted on x-axis and true gesture labels on y-axis which return accuracy rate value using SVM on the affected side: (**a**) all possible combinations (20) withing 4 gesture labels (GL4) with the total mean accuracy rate of 90.07%; (**b**) all possible combinations (15) withing 5 gesture labels (GL5) with the total mean accuracy rate of 89.24%; (**c**) all possible combinations (6) withing 6 gesture labels (GL6) with the total mean accuracy rate of 88.91%; (**d**) one possible combination withing 7 gesture labels (GL7) with the total mean accuracy rate of 89.75%. PCA has not been applied for ASF-24P feature set.

Confusion matrix charts of possible random gesture combinations in P19 using LDA.

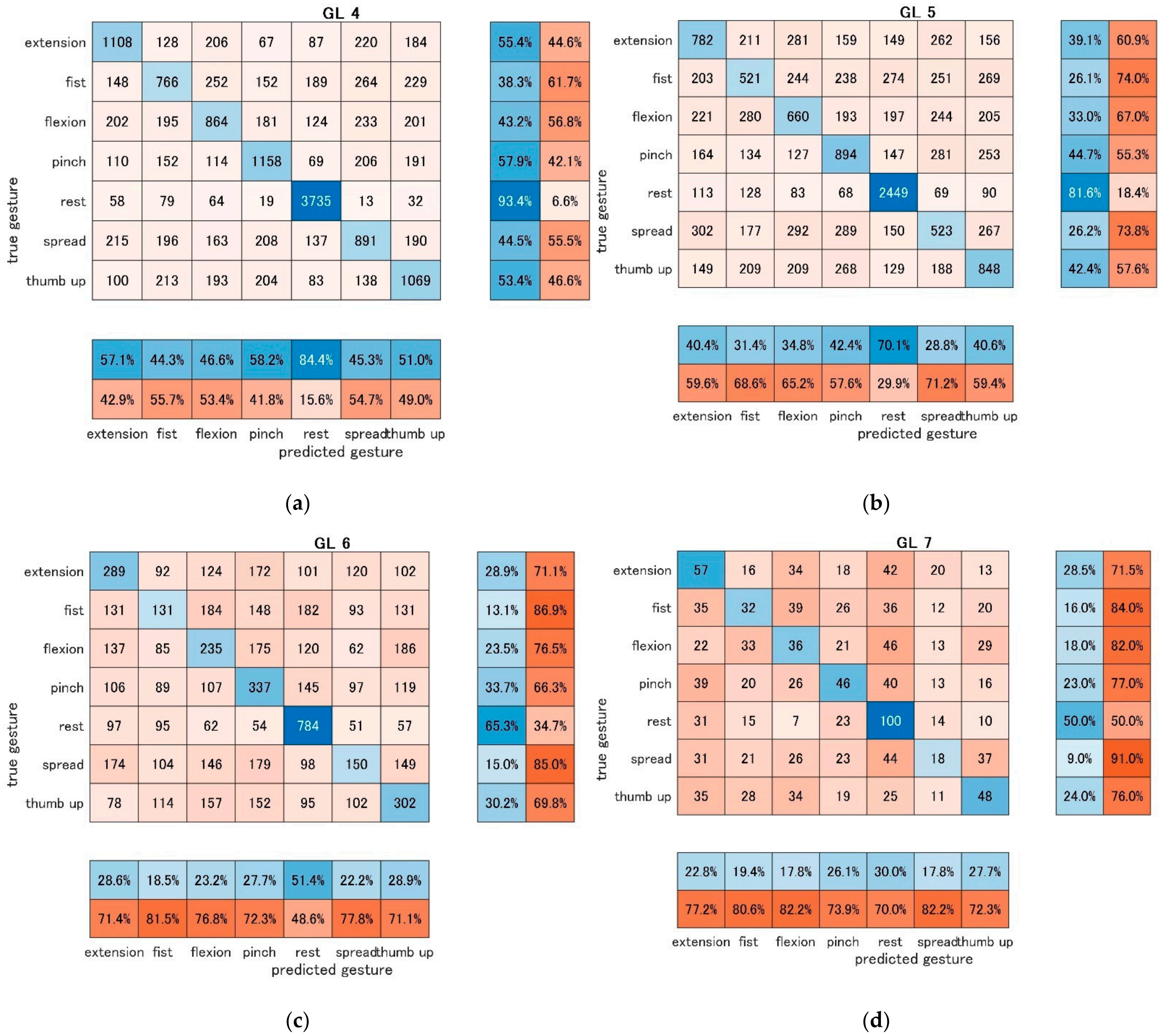

Figure S6. This is paretic cross-validation confusion matrix charts with predicted on x-axis and true gesture labels on y-axis which return accuracy rate value using LDA on the affected side: (**a**) all possible combinations (20) withing 4 gesture labels (GL4) with the total mean accuracy rate of 80.17%; (**b**) all possible combinations (15) withing 5 gesture labels (GL5) with the total mean accuracy rate of 77.89%; (**c**) all possible combinations (6) withing 6 gesture labels (GL6) with the total mean accuracy rate of 77.60%; (**d**) one possible combination withing 7 gesture labels (GL7) with the total mean accuracy rate of 77.42%. PCA has not been applied for ASF-24P feature set.

Confusion matrix charts of possible random gesture combinations in P19 using k-NN.

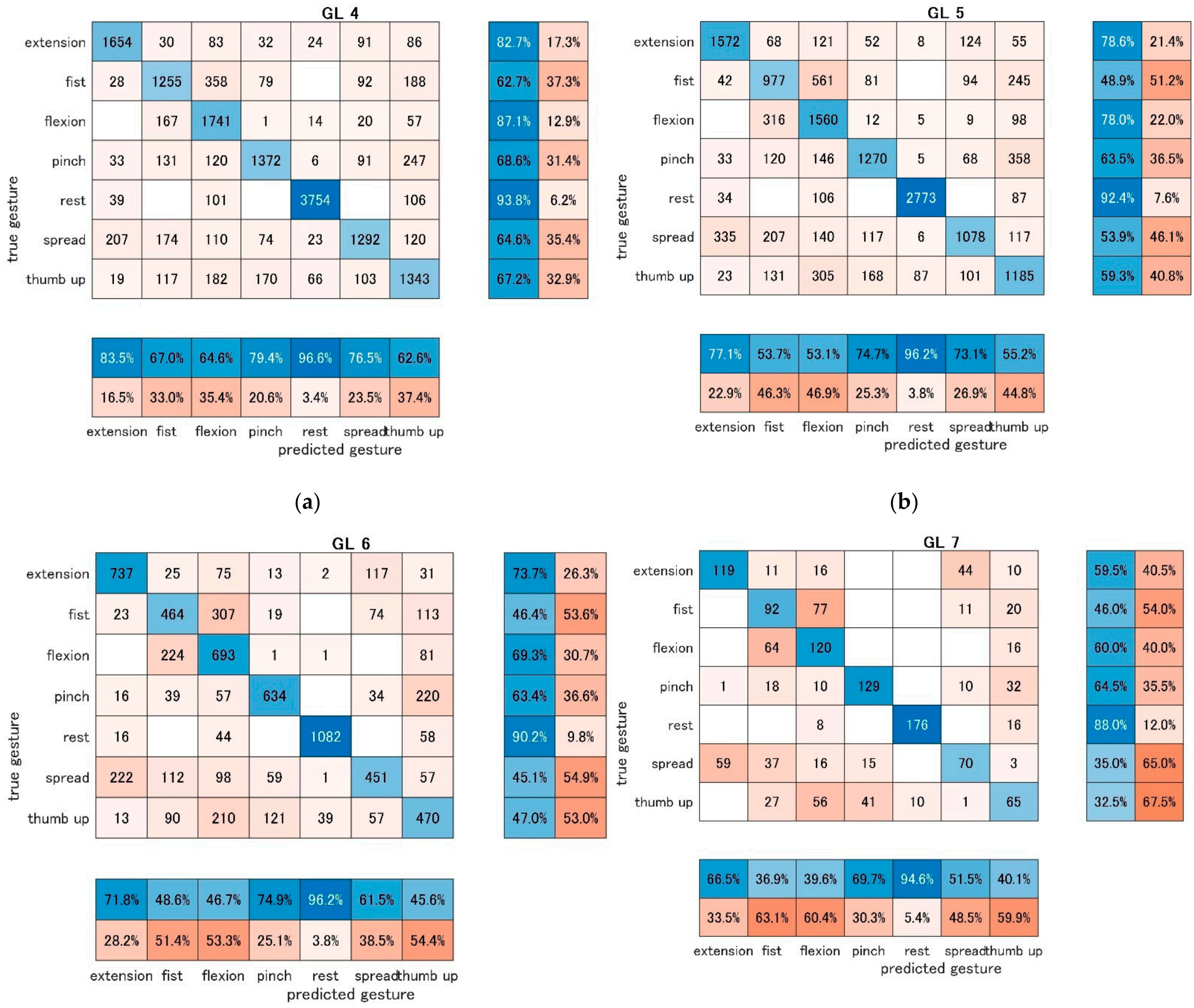

(a) (b) (c) (d)

Figure S7. This is paretic cross-validation confusion matrix charts with predicted on x-axis and true gesture labels on y-axis which return accuracy rate value using k-NN on the affected side: (**a**) all possible combinations (20) withing 4 gesture labels (GL4) with the total mean accuracy rate of 88.56%; (**b**) all possible combinations (15) withing 5 gesture labels (GL5) with the total mean accuracy rate of 88.08%; (**c**) all possible combinations (6) withing 6 gesture labels (GL6) with the total mean accuracy rate of 87.10%; (**d**) one possible combination withing 7 gesture labels (GL7) with the total mean accuracy rate of 87.30%. PCA has not been applied for ASF-24P feature set.

Confusion matrix charts of possible random gesture combinations in NP19 using SVM with the dimensional reduction using PCA.

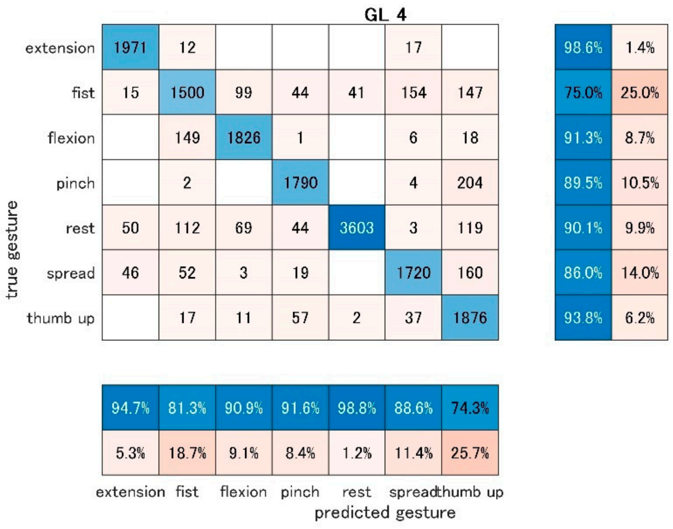

(**a**)

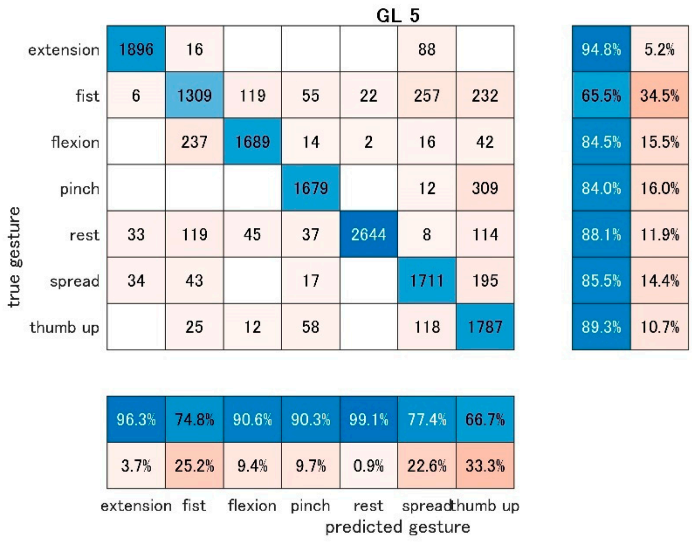

(**b**)

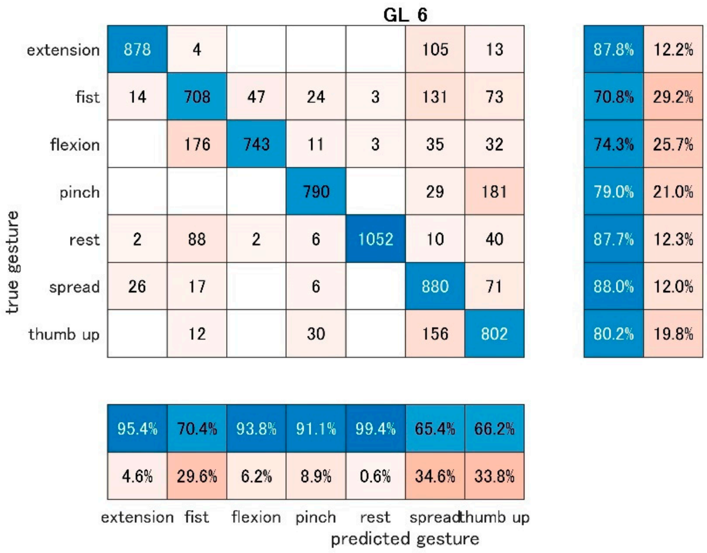

(**c**)

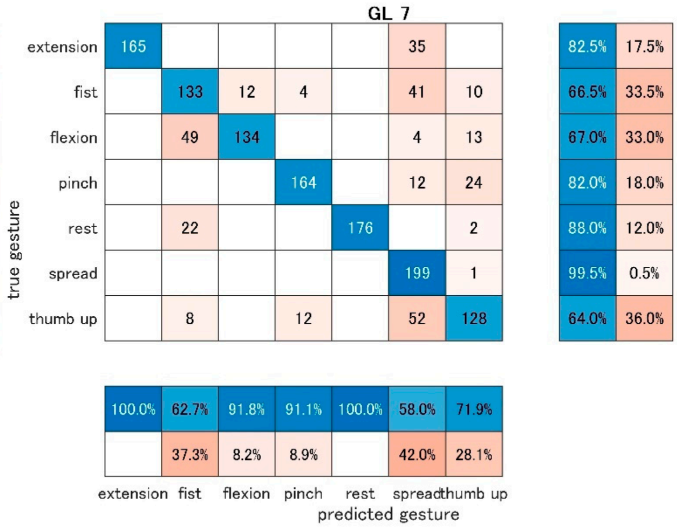

(**d**)

Figure S8. This is non-paretic cross-validation confusion matrix charts with predicted on x-axis and true gesture labels on y-axis which return accuracy rate value using SVM on the non-affected side: (**a**) all possible combinations (20) withing 4 gesture labels (GL4) with the total mean accuracy rate of 94.81%; (**b**) all possible combinations (15) withing 5 gesture labels (GL5) with the total mean accuracy rate of 94.00%; (**c**) all possible combinations (6) withing 6 gesture labels (GL6) with the total mean accuracy rate of 93.82%; (**d**) one possible combination withing 7 gesture labels (GL7) with the total mean accuracy rate of 93.53%. PCA set to 23 at the ASF-14NP feature set.

Confusion matrix charts of possible random gesture combinations in NP19 using LDA with the dimensional reduction using PCA.

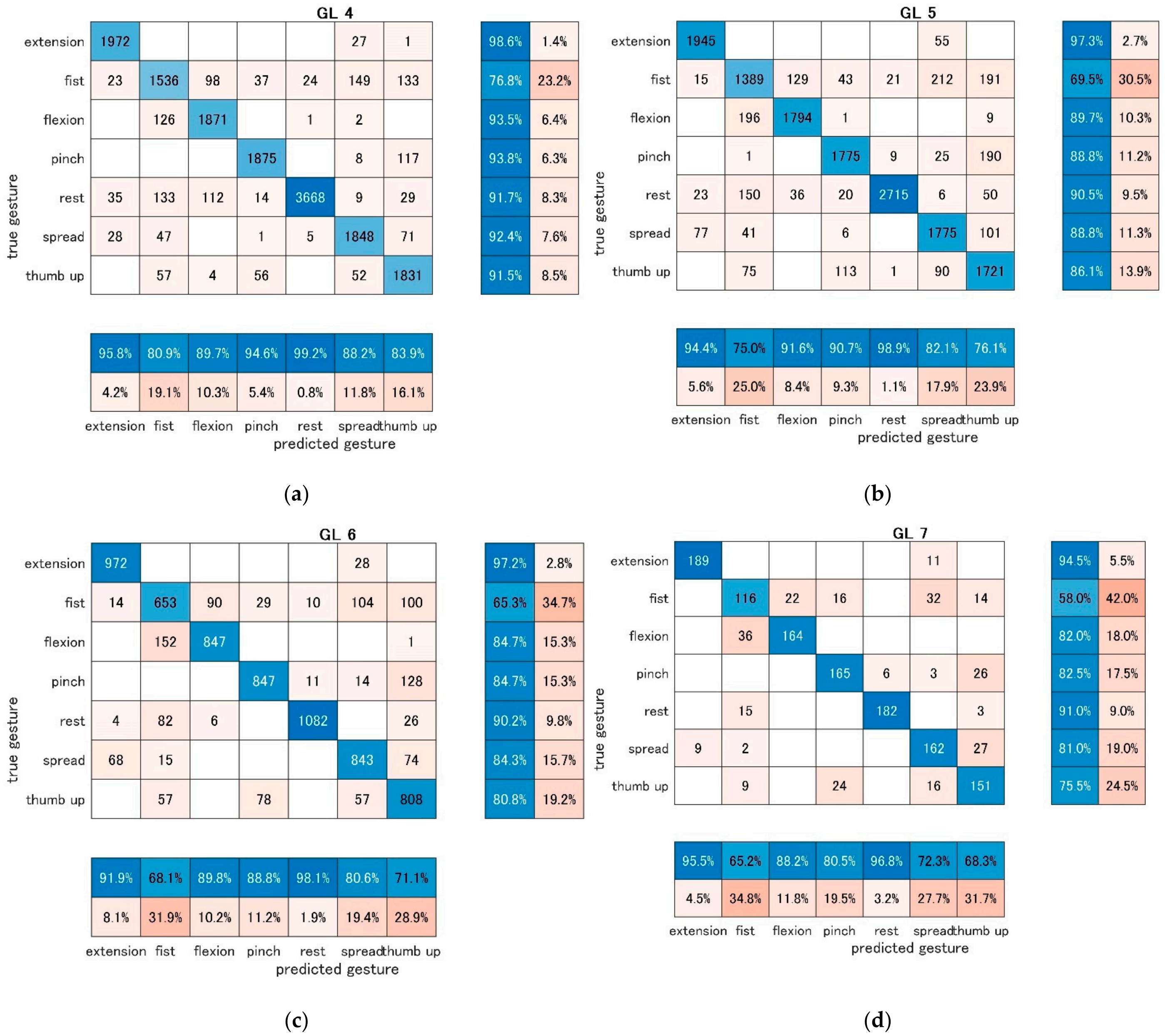

Figure S9. This is non-paretic cross-validation confusion matrix charts with predicted on x-axis and true gesture labels on y-axis which return accuracy rate value using LDA on the non-affected side: (**a**) all possible combinations (20) withing 4 gesture labels (GL4) with the total mean accuracy rate of 95.42%; (**b**) all possible combinations (15) withing 5 gesture labels (GL5) with the total mean accuracy rate of 95.30%; (**c**) all possible combinations (6) withing 6 gesture labels (GL6) with the total mean accuracy rate of 94.81%; (**d**) one possible combination withing 7 gesture labels (GL7) with the total mean accuracy rate of 94.36%. PCA set to 19 at the ASF-14NP feature set.

Confusion matrix charts of possible random gesture combinations in NP19 using k-NN with the dimensional reduction using PCA.

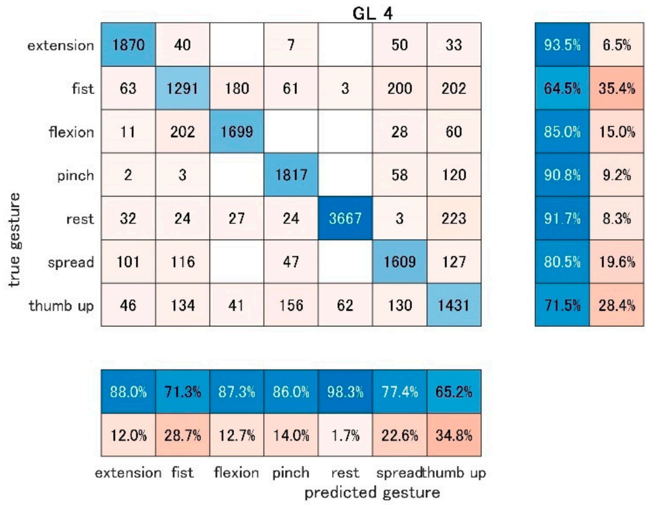

(**a**)

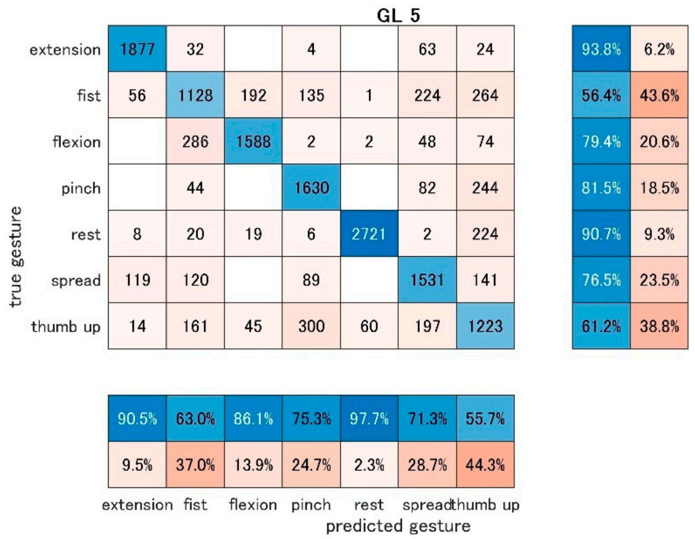

(**b**)

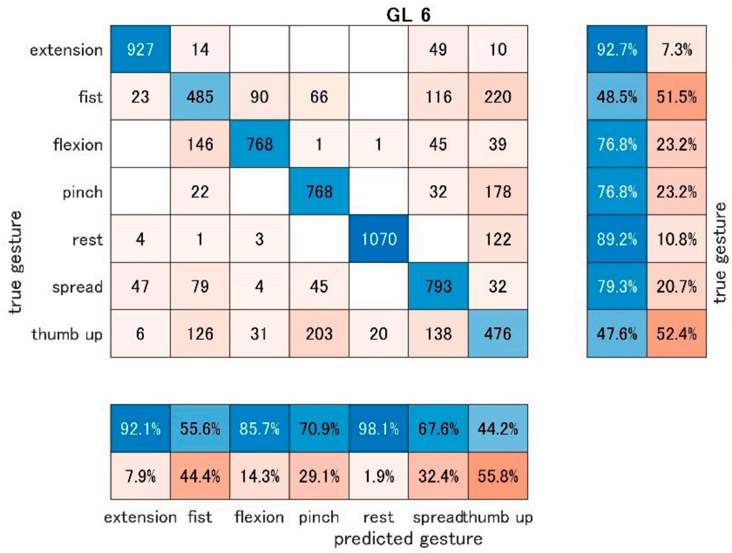

(**c**)

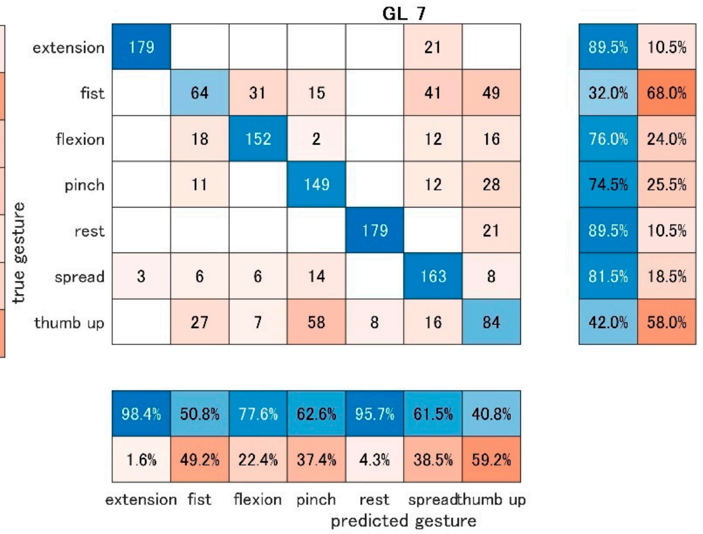

(**d**)

Figure S10. This is non-paretic cross-validation confusion matrix charts with predicted on x-axis and true gesture labels on y-axis which return accuracy rate value using k-NN on the non-affected side: (**a**) all possible combinations (20) withing 4 gesture labels (GL4) with the total mean accuracy rate of 92.15%; (**b**) all possible combinations (15) withing 5 gesture labels (GL5) with the total mean accuracy rate of 91.63%; (**c**) all possible combinations (6) withing 6 gesture labels (GL6) with the total mean accuracy rate of 91.63%; (**d**) one possible combination withing 7 gesture labels (GL7) with the total mean accuracy rate of 90.83%. PCA set to 8 at the ASF-14NP feature set.

Confusion matrix charts of possible random gesture combinations in P19 using SVM with the dimensional reduction using PCA.

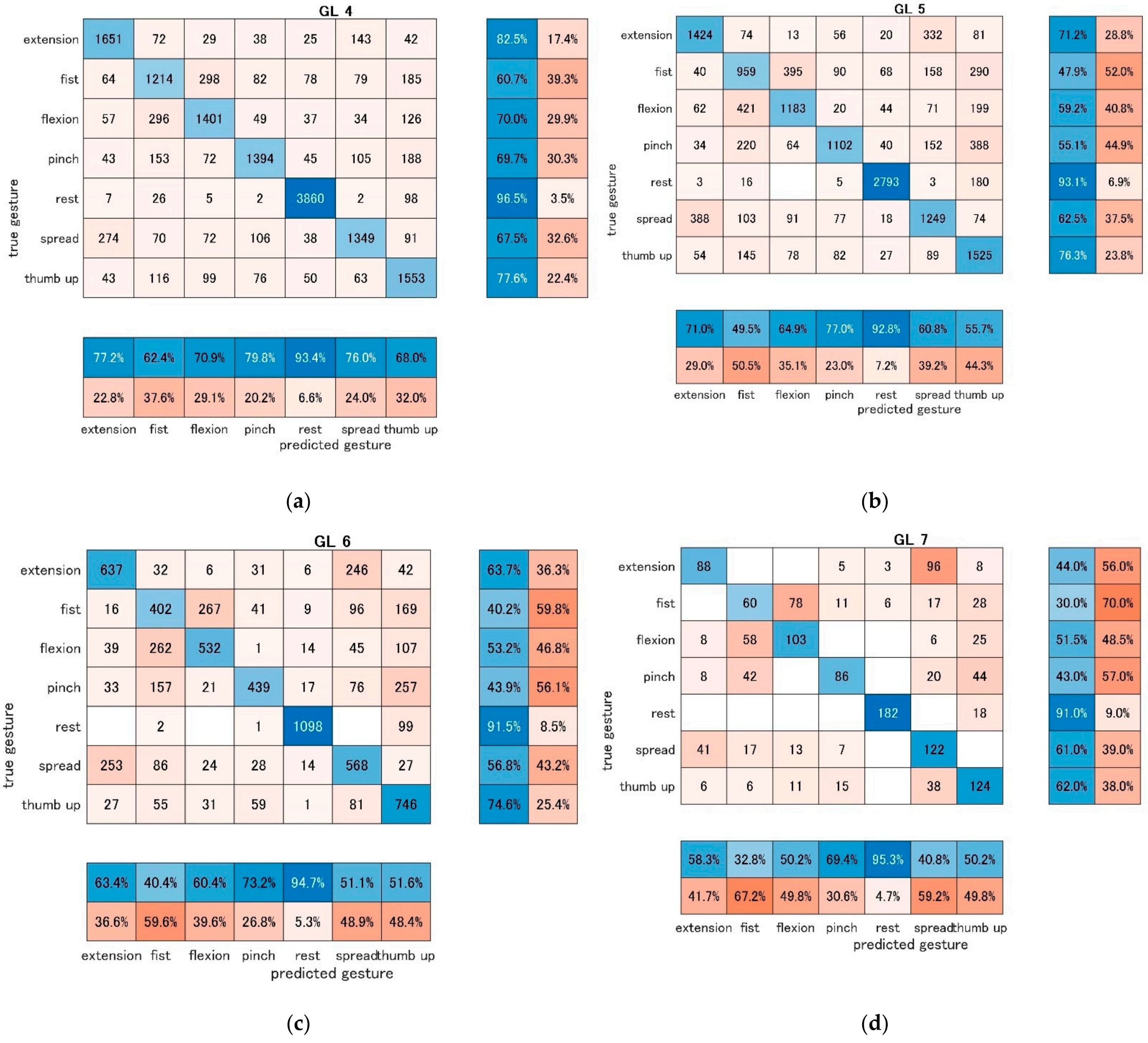

Figure S11. This is paretic cross-validation confusion matrix charts with predicted on x-axis and true gesture labels on y-axis which return accuracy rate value using SVM on the affected side: (**a**) all possible combinations (20) withing 4 gesture labels (GL4) with the total mean accuracy rate of 88.67%; (**b**) all possible combinations (15) withing 5 gesture labels (GL5) with the total mean accuracy rate of 87.45%; (**c**) all possible combinations (6) withing 6 gesture labels (GL6) with the total mean accuracy rate of 87.13%; (**d**) one possible combination withing 7 gesture labels (GL7) with the total mean accuracy rate of 87.30%. PCA set to 35 at the ASF-24P feature set.

Confusion matrix charts of possible random gesture combinations in P19 using LDA with the dimensional reduction using PCA.

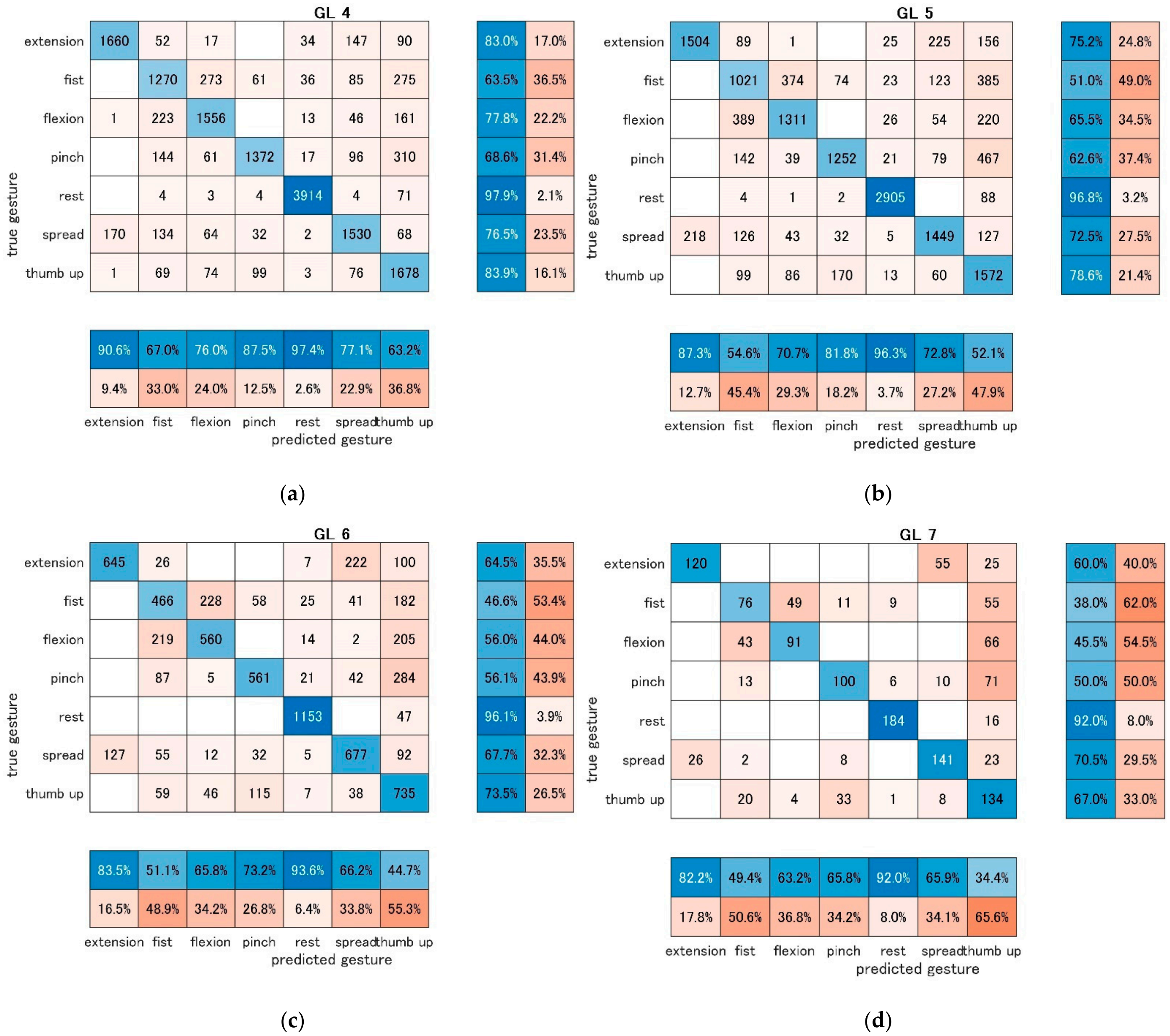

Figure S12. This is paretic cross-validation confusion matrix charts with predicted on x-axis and true gesture labels on y-axis which return accuracy rate value using LDA on the affected side: (**a**) all possible combinations (20) withing 4 gesture labels (GL4) with the total mean accuracy rate of 90.20%; (**b**) all possible combinations (15) withing 5 gesture labels (GL5) with the total mean accuracy rate of 89.57%; (**c**) all possible combinations (6) withing 6 gesture labels (GL6) with the total mean accuracy rate of 89.32%; (**d**) one possible combination withing 7 gesture labels (GL7) with the total mean accuracy rate of 89.27%. PCA set to 5 at the ASF-24P feature set.

Confusion matrix charts of possible random gesture combinations in P19 using k-NN with the dimensional reduction using PCA.

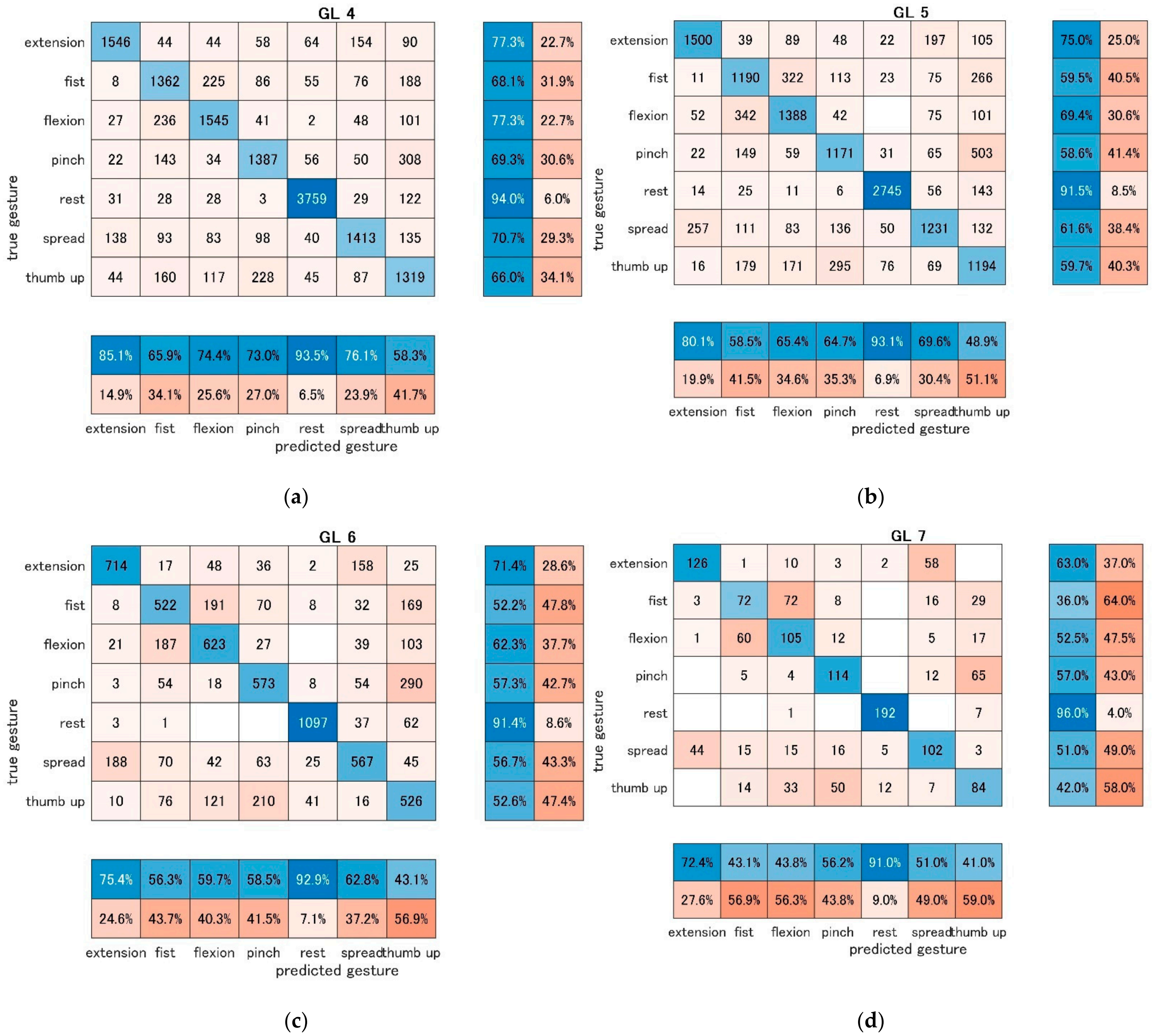

Figure S13. This is paretic cross-validation confusion matrix charts with predicted on x-axis and true gesture labels on y-axis which return accuracy rate value using k-NN on the affected side: (**a**) all possible combinations (20) withing 4 gesture labels (GL4) with the total mean accuracy rate of 88.55%; (**b**) all possible combinations (15) withing 5 gesture labels (GL5) with the total mean accuracy rate of 87.98%; (**c**) all possible combinations (6) withing 6 gesture labels (GL6) with the total mean accuracy rate of 87.35%; (**d**) one possible combination withing 7 gesture labels (GL7) with the total mean accuracy rate of 88.88%. PCA set to 5 at the ASF-24P feature set.

Table S1. Statistical verification of max of mean accuracy obtained by SVM, LDA, and k-NN with and without the use of PCA.

Number of Gesture Labels	No PCA	PCA Dim. 5	PCA Dim. 8	PCA Dim. 19	PCA Dim. 23	PCA Dim 35
GL4 (P)	SVM *	LDA*	LDA*	LDA*	LDA*	LDA
GL4 (NP)	SVM*	LDA*	LDA*	LDA*	LDA*	LDA*
GL5 (P)	SVM*	LDA*	LDA*	LDA*	LDA*	LDA*
GL5 (NP)	SVM*	LDA*	LDA*	LDA*	LDA*	LDA*
GL6 (P)	SVM*	LDA*	LDA*	LDA*	LDA*	LDA*
GL6 (NP)	SVM*	LDA*	LDA*	LDA*	LDA*	LDA*
GL7 (P)	SVM	LDA	LDA	LDA	LDA	LDA
GL7 (NP)	SVM	LDA*	LDA*	LDA	LDA	LDA

* Asterisk indicates statistical significance based on the paired *t*-test ($p < 0.05$).

Table S2. Statistical verification of max F_1-score performance metrics obtained by SVM, LDA, and k-NN with and without the use of PCA.

Number of Gesture Labels	No PCA	PCA Dim. 5	PCA Dim. 8	PCA Dim. 19	PCA Dim. 23	PCA Dim 35
GL4 (P)	SVM *	LDA*	LDA*	LDA*	LDA*	LDA
GL4 (NP)	SVM*	LDA*	LDA*	LDA*	LDA*	LDA*
GL5 (P)	SVM*	LDA*	LDA*	LDA*	LDA*	LDA*
GL5 (NP)	SVM*	LDA*	LDA*	LDA*	LDA*	LDA*
GL6 (P)	SVM*	LDA*	LDA*	LDA*	LDA*	LDA*
GL6 (NP)	SVM*	LDA*	LDA*	LDA*	LDA*	LDA*
GL7 (P)	SVM	LDA*	LDA	LDA	LDA	LDA
GL7 (NP)	SVM	LDA*	LDA*	LDA	LDA	LDA

* Asterisk indicates statistical significance based on the paired *t*-test ($p < 0.05$).
